# Development and therapeutic applications of nitric oxide-releasing materials

**DOI:** 10.4155/FSO.15.50

**Published:** 2015-08-01

**Authors:** Joel M Friedman, Adam J Friedman

**Affiliations:** 1 Department of Physiology & Biophysics, Albert Einstein College of Medicine, Bronx, NY 10461, USA; 2 Montefiore – Albert Einstein College of Medicine, 111 E 210th Street, Bronx, NY 10467, USA; 3 George Washington School of Medicine & Health Sciences, Washington, DC 20052, USA

**Keywords:** drug delivery, drug development, nitric oxide

Prior to the truly seismic revelation that nitric oxide (NO) was the elusive endothelium-derived relaxing factor, NO was considered simply a reactive pollutant for the vast majority of the biology and biomedical research community – there was essentially no linkage to actual *in vivo* functional processes, let alone therapeutic applications. Even the relationship between NO and the therapeutic efficacy of nitroglycerin was at this point still unknown. The abrupt change in the research status of NO, initiated by the monumental discovery of the NO-elusive endothelium-derived relaxing factor connection, heralded a new era of investigation that continues to dramatically expand every year. Research on NO has now expanded to include: mechanisms of physiological and biochemical action; the importance of temporal and site-specific NO generation under both normal and pathological conditions; the nature, properties and synthesis of longer lived NO-related molecules that preserve and extend NO bioactivity; the role of nitrite as a stable source of physiological levels of NO; potential therapeutic targets for NO and other NO-related molecules; food and supplements to enhance levels of NO in the circulation; and development of topical and systemic delivery vehicles for therapeutic levels of NO and NO-releasing molecules. In fact, it is becoming ever more apparent that many of the current drugs being developed to address numerous disease conditions are in effect elegant methods of stimulating NO in the appropriate locations and amount. What is also clear is with every new discovery and shift of the research landscape, new NO-related questions emerge at an equally steady pace. Issues seemingly as fundamental as to whether NO promotes or inhibits cancer growth remain controversial. Limitations in addressing not only basic questions relating to how NO participates in both physiological and pathophysiological processes but also how to utilize NO therapeutically arise from an inability to appropriately and effectively deliver and monitor NO *in vivo*. This issue is an attempt to update overviews of where we are with respect to several of these rapidly evolving NO associated foci of research with an emphasis on delivery and detection strategies. We are fully cognizant that with the level of activity and the rate at which new insights are emerging, we can at best provide but a transient depiction that represents at least a breath catching pause for researchers to take stock of what has been accomplished and where we might be headed. The contributors to this effort are all at the cutting edge of NO research and represent a cohesive effort to encompass many of the important issues that pertain to the ultimate biomedical objective of harnessing the extraordinary therapeutic potential of this seemingly simple molecule.

